# Bone mineral density in patients diagnosed with giant cell arteritis taking glucocorticoids: a case–control study

**DOI:** 10.1093/rap/rkad020

**Published:** 2023-02-21

**Authors:** Adam Geressu, Zain Sultan, Marwan Bukhari

**Affiliations:** University Hospitals of Morecambe Bay NHS Foundation Trust, Royal Lancaster Infirmary, Lancaster, UK; University Hospitals of Morecambe Bay NHS Foundation Trust, Royal Lancaster Infirmary, Lancaster, UK; Rheumatology Department, Faculty of Health Sciences, University of Manchester, Manchester, UK

**Keywords:** BMD, GCA, glucocorticoids, ophthalmology

## Abstract

**Objective:**

The standard treatment for GCA is high-dose glucocorticoids (GCs). It is unknown whether GCs are more detrimental to BMD at the spine or the hip. The aim of this study was to investigate the effect of GCs on BMD at the lumbar spine and hip in patients with GCA being treated with GCs.

**Methods:**

Patients who were referred for DXA at a hospital in the north-west of England between 2010 and 2019 were included. Two patient groups were identified: patients with GCA on current GC (cases) were matched 1:4 based on age and biological sex to those referred to the scanner with no indication for scanning (controls). Logistic models were fitted looking at the spine and hip BMD, unadjusted and adjusted for height and weight.

**Results:**

As would be expected, this gave an adjusted odds ratio (OR) of 0.280 (95% CI 0.071, 1.110) at the lumbar spine, OR of 0.238 (95% CI 0.033, 1.719) at the left femoral neck, OR of 0.187 (95% CI 0.037, 0.948) at the right femoral neck, OR of 0.005 (95% CI 0.001, 0.021) at the left total hip and OR of 0.003 (95% CI 0.001, 0.015) at the right total hip.

**Conclusion:**

The study has shown that patients diagnosed with GCA receiving GC treatment have a lower BMD at the right femoral neck, left total hip and right total hip compared with controls in patients of the same age and biological sex after adjusting for height and weight.

Key messages

- Patients diagnosed with GCA taking glucocorticoids have lower BMD at specific body sites.
- Glucocorticoids have been found to be associated with lower hip BMD.

## Introduction

GCA is the commonest type of vasculitis affecting medium and large vessels [1]. GCA affects those >50 years of age almost exclusively. The incidence of GCA in people >50 years old is ∼18 per 100 000 per year [2]. Glucocorticoids (GCs) are the mainstay of treatment for GCA. GCs when given early are most effective in treating and preventing the serious ophthalmic complications of GCA [3].

To date, there has been a mixture of results on the impact of GCs on BMD in GCA. Haugeberg *et al.* [4] found that BMD was not significantly reduced in patients with GCA who were either previously or currently taking low-dose prednisolone in comparison to newly diagnosed patients who were yet to commence GC treatment. However, Emamifar *et al.* [5] found that treatment with GCs in patients with GCA caused a decreased in BMC and BMD.

Most studies that have investigated the impact of GC on BMD have focused on the impact of general bone loss of both trabecular (hip) and cortical (spine) bone, as opposed to specific body locations. In our study, we used a larger sample size of patients compared with other similar studies, in order to increase the power of the study. The aim of our study was to assess whether GC use in patients diagnosed with GCA causes a decrease in BMD and, if so, whether it is more detrimental to the spine or the hip.

## Methods

### Participants

Sequential patients diagnosed with GCA referred for DXA to a single scanner in the north-west of England between 2010 and 2019 were compared 1:4 with patients referred with no indication for DXA scanning. The patients in the control group were those who visited their general practitioner with concerns about their BMD and who were sent for DXA scanning. Patients were scanned with a GE Lunar iDXA. Demographic data collected at the time of scanning are shown in Table 1.

**Table 1.** Baseline characteristics for all cases and controls

Baseline characteristics	Cases (*n* = 62)	Controls (*n* = 248)	*P*-value
Age, years			
Mean (s.d.)	71.6 (7.4)	72.1 (7.7)	0.6487
50–70	21 (34)	84 (34)	
>70	41 (66)	164 (66)	
Biological sex			1.0000
Male	13 (21)	52 (21)	
Female	49 (79)	196 (79)	
Height, m			
Mean (s.d.)	163.1 (7.0)	161.3 (8.5)	0.1221
<1.6	13 (21)	122 (49)	
>1.6	49 (79)	126 (51)	
Weight, kg			
Mean (s.d.)	74.5 (17.0)	70.7 (14.5)	0.0785
<50	1 (1.5)	13 (5)	
50–100	57 (92)	225 (91)	
>100	4 (6.5)	10 (4)	
BMI, kg/m^2^			
Mean (s.d.)	28.0 (5.7)	27.2 (5.1)	0.2907
<18.5	0 (0)	4 (2)	
18.5–24.9	22 (35)	58 (23)	
25–29.9	22 (35)	107 (43)	
>30	18 (30)	79 (32)	
BMD, mean (s.d.), g/cm^2^			
Lumbar spine	1.047	1.084	0.264
Left femoral neck	0.816	0.831	0.482
Right femoral neck	0.801	0.831	0.226
Left total hip	0.386	0.878	0.000
Right total hip	0.375	0.884	0.000

Values are the number (percentage) unless indicated otherwise.

We recorded the BMD (in grams per square metre) at the lumbar spine (mean of L1–L4 vertebrae), left femoral neck, right femoral neck, left total hip and right total hip, in addition to their corresponding T-scores (comparison of a person's bone density with that of a healthy 30-year-old of the same sex) and Z-scores (comparison of a person's bone density with that of an average person of the same age and sex). We collected data on the patient’s age at scanning, biological sex, height and weight. We also gathered data on multiple risk factors, such as smoking, excess alcohol use, history of fractures, family history of fractures/parental history of hip fractures, CS use, osteoporosis and RA. In addition, we collected data on current or past osteoporosis treatment that patients were receiving at the time of DXA, which included CSs, proton pump inhibitors, risedronate, zoledronate, calcium, a combination of calcium and vitamin D, vitamin D, alendronate, clodronate, ibandronic acid, pamidronate and raloxifene. The patients did not give written consent, but the data were pseudonymized and the study was passed by the Northwest ethics committee (reference number: 21/NW/0309).

### Statistical analysis

Initially, cases and controls were compared using Student’s *t*-test for continuous variables and the χ^2^ test for categorical variables. Subsequently, logistic models were fitted, both unadjusted and adjusted for height and weight, looking at the odds of a low BMD at the lumbar spine, right femoral neck, left femoral neck, left total hip and right total hip. The data were analysed using STATA for Windows v.13.0.

## Results

In this study, there was a total of 310 patients, 62 of whom were cases and 248 of whom were controls. There were no patients in the study population <50 years of age. Baseline characteristics of the cases and controls are shown in Table 1. There was no significant difference in baseline characteristics. Both height and weight were adjusted for in the case and control groups. We also looked at risk factors in our 62 cases. There were 25 patients who were smokers, of whom 7 were current smokers at the time of DXA. There were six patients who drank excess alcohol. There were three patients who were diagnosed with RA. There were no patients who had secondary osteoporosis. There were 17 patients who had a history of fractures. There were 16 patients who had a family history of fractures, of whom 2 patients had a history of hip fracture in their mother or father. There were 18 patients who used CSs, of whom 11 patients were using CSs at time of DXA. In terms of medications, out of the 62 patients, 0 were taking proton pump inhibitors, 2 were taking risedronate, 1 was taking zoldronate, 6 were taking alendronate, 0 were taking clodronate, 0 were taking pamidronate, 0 were taking ibandronic acid, 0 were taking raloxifene, 0 were taking calcium alone, 0 were taking vitamin D alone, and 16 were taking a combination of calcium and vitamin D. In the control group of 248 patients, there were none of the additional risk factors reported in the cases.

**Figure 1.**
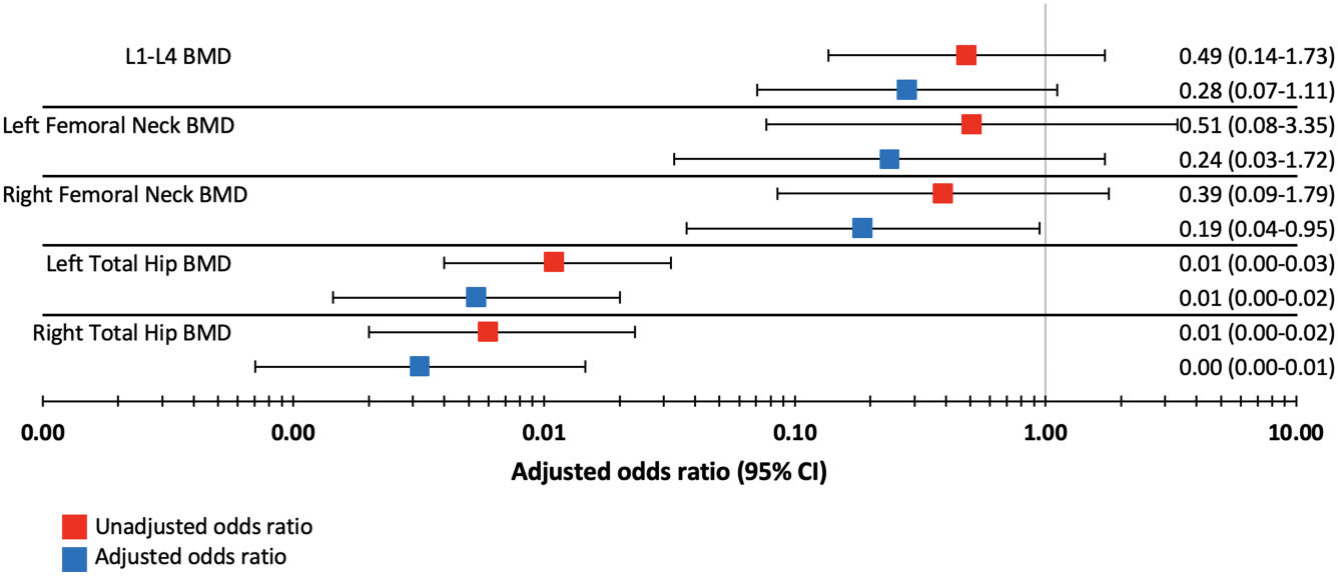
The results of five logistic regression models showing the impact of BMD on the odds of having GCA receiving glucocorticoid (GC) at different body locations, all adjusted for height and weight

Patients diagnosed with GCA receiving GCs had lower values of BMD than patients not diagnosed with GCA who were of the same age and biological sex after adjusting for height and weight. Fig. 1 shows the results of the adjusted and unadjusted logistic regression. There was an odds ratio (OR) of 0.280 (95% CI 0.071, 1.110) at the lumbar spine, OR of 0.238 (95% CI 0.033, 1.719) at the left femoral neck, OR of 0.187 (95% CI 0.037, 0.948) at the right femoral neck, OR of 0.005 (95% CI 0.001, 0.021) at the left total hip and OR of 0.003 (95% CI 0.001, 0.015) at the right total hip. The odds of having GCA with GC compared with those who did not have GCA decreased by 0.19 (95% CI 0.04, 0.95) per unit change in BMD at the right femoral neck location. At the left total hip location, the odds decreased by 0.005 (95% CI 0.001, 0.021), and at the right total hip location it decreased by 0.003 (95% CI 0.001, 0.015) per unit change in BMD. There was no statistical evidence of an association between BMD and the odds of having GCA with GCs at the lumbar spine and left femoral neck locations.

## Discussion

### What was found and how does this differ from the literature?

Our study revealed patients with GCA treated with GCs have significantly lower BMD in the right femoral neck, left total hip and right total hip when compared with controls. BMD values were also lower for the spine and the left femoral neck in patients with GCA on GCs when compared with controls; however, they were not statistically significant. There have been many publications outlining the multifaceted effect of GCs on bone remodelling [6]. In line with our findings, Emamifar *et al.* [5] studied patients with PMR and GCA who were given high-dose prednisolone. DXA scans done at baseline and at week 40 found that patients had a significantly reduced total BMC [5]. Hatz *et al.* [7] also reported the main side effect of osteoporosis in an open prospective study involving 47 patients who took 10 mg prednisolone over 6 months for the treatment of GCA or PMR.

Contrary to our results, Haugeberg *et al.* [4] found that BMD was not significantly reduced in patients with GCA and PMR who were either previously or currently taking low-dose prednisolone, in comparison to newly diagnosed patients who were yet to commence GC treatment. The GC dose used in their study was much lower (6.5 mg) [4] than what is advised by the National Institute for Health and Care Excellence (NICE) in the UK (40–60 mg) [8], which might be why the BMD was not significantly reduced. The control group used in that study were diagnosed with GCA and PMR but had not yet started GC therapy, hence the lack of difference in BMD between cases and controls might be accounted for by the fact that the inflammatory condition itself reduces BMD. In fact, inflammatory processes in other autoimmune conditions, such as RA, have been shown to impact bone density negatively [9]. Through our study and a review of literature, it is unclear whether the reduction in BMD in GCA patients taking GCs is attributable to the disease or the treatment, or a combination. Data on this topic are limited, but it has been reported that generally, it is a combination of the inflammatory process and GC therapy that leads to a reduction in BMD [5]. Ascertaining the true difference in effect on BMD by the inflammatory process alone (without GC treatment) *vs* the effect on BMD of GC treatment in the setting of GCA is neither ethical nor possible owing to the severe consequences to the patients of untreated GCA.

Our study results lead us to question whether some areas or types of bone are affected more than others by GC. In an older study from 1998, Pearce *et al.* [10] examined the change in BMD in patients with PMR who took 10 mg prednisolone daily for 14 months.They found a statistically significant overall reduction in total body bone mass, which agrees with our results. They also presented a reduction of BMD in different regions of the body as follows: lumbar spine (2.6 ± 0.8%, *P* < 0.01); femoral neck (2.9 ± 1.5%, *P* = 0.06); Ward’s triangle (5.5 ± 2.9%, *P* = 0.06); and the trochanter (4.3 ± 1.9%, *P* < 0.05) [10], thus trabecular and cortical bone are both affected by GC use in PMR, essentially stating that bone loss is general [10]. We have questioned in our study whether GC is more detrimental to the spine (relatively more trabecular bone present) or hip (relatively more cortical bone present). Our study suggests that patients on GCs for GCA had a statistically lower BMD in the hip when compared with controls.

### Strengths of the study

This study has strengths and limitations. The strengths include matching cases and controls by age and biological sex, in addition to adjusting for height and weight in order to reduce the effect of confounding variables and improving the reliability of our results. Another strength is that we have a large sample size of 310 patients, which increases the power of the study.

### Weaknesses

However, our retrospective case–control study was not without some limitations. Some confounding variables, such as patient use of pharmacological bone protection and risk factors for osteoporosis (e.g. smoking history), were not taken into account. We do not have information on the dose or duration of GC treatment and at what point the DXA measurement was done in the course of the disease. Patients did not receive a DXA scan at the start of GC treatment and then during/after GC treatment, thus the intra-subject variability in BMD before and after the exposure to GC was not examined. Furthermore, the additional risk factors reported by patients in the case group were not matched for in the control group.

### Conclusion

In conclusion, this retrospective case–control study of a large cohort indicated that patients of the same age and biological sex diagnosed with GCA using GCs have a lower BMD than controls at the right femoral neck, left total hip and right total hip, after adjusting for height and weight. There is no statistical evidence of an association between BMD and the odds of having GCA with GCs at the lumber spine and left femoral neck locations. This suggests that, overall, GCs have been associated with lower hip BMD. These findings should be of value when clinicians are prescribing GCs in patients diagnosed with GCA and provide a basis for future considerations, particularly guidance surrounding bone protection in GCA and future avenues of research. A useful future avenue of research would be to investigate the incidence of fractures in patients with GCA and taking GCs.

## Data Availability

The data underlying this article will be shared on reasonable request to the corresponding author.
